# Carbon Nanotubes Act as Contaminant Carriers and Translocate within Plants

**DOI:** 10.1038/srep15682

**Published:** 2015-10-26

**Authors:** Guosheng Chen, Junlang Qiu, Yan Liu, Ruifen Jiang, Siying Cai, Yuan Liu, Fang Zhu, Feng Zeng, Tiangang Luan, Gangfeng Ouyang

**Affiliations:** 1 MOE Key Laboratory of Aquatic Product Safety/KLGHEI of Environment and Energy Chemistry, School of Chemistry and Chemical Engineering, Sun Yat-sen University, Guangzhou 510275, China; 2 Department of Food Science and Technology, College of Food Science and Technology, Shanghai Ocean University, Shanghai 201306, China.

## Abstract

Nanotechnology permits broad advances in agriculture. However, as it is still at a relatively early stage of development, the potential risks remain unclear. Herein, for the first time, we reveal the following: 1) the impact of multi-walled carbon nanotubes (MWCNTs) on the accumulation/depuration behaviors of contaminants in crop, mustard (*Brassica juncea*), and 2) the permeability and transportability of MWCNTs in intact mature mustard plants. Using an *in vivo* sampling technique, the kinetic accumulation/depuration processes of several contaminants in mustard plans exposed to MWCNTs were traced, and an enhancement of contaminant accumulation in living plants was observed. Meanwhile, we observed that the MWCNTs permeated into the roots of intact living plants (three months old) and were then transported to the upper organs under the force of transpiration steam. This study demonstrated that MWCNTs can act as contaminant carriers and be transported to the edible parts of crops.

Nanoscience is one of the most important research and development frontiers in modern science^1^,^2^,^3^. Carbon nanotubes (CNTs), with their combination of a microtubular structure and particular physical properties^4^, are promising materials for biotechnology applications^5^,^6^,^7^. CNTs also act as transporters to permeate into tumor cells^8^, bacteria^9^, plant cells^10^, and animal tissues^11^, which opened a new route for drug and gene delivery. In addition, there is extensive interest in applying CNTs to plants for agricultural use^12^, and exciting achievements such as seed germination enhancement, root growth, and an increase in biomass have been reported^13^,^14^,^15^,^16^. However, the fact is that nanotechnology is still in a relative early stage of development, which inevitably arouses consideration about the environmental, health, and safety impacts of the use of CNTs in agriculture^17^. Investigations have shown that CNTs could induce phytotoxicity in plant cells^18^ and change the gene expression of plants^10^. These studies indicated that comprehensive assessment and understanding of the potential risks of the application of CNTs in agriculture are critical to achieving the goals of “nano-agriculture.” For example, a recent study proved that a rice autologous transporter can reduce the accumulation of a highly toxic metalloid, arsenic, in the grain^19^. However, to the best of our knowledge, the effects of CNTs on the enrichment/depuration of contaminants in crops are unclear, and more importantly, the permeability of CNTs in intact mature plants as well as the translocation of CNTs within plants have not explored. If the CNTs can penetrate the cell walls of plants and translocate to the edible parts of crops, they will enter the food chain and cause a risk to human health.

To investigate the interactions between CNTs and contaminants in crops, the sampling technology must satisfy the following conditions: 1) to ensure that the same plant samples can be simultaneously used for analysis of the contaminants and CNTs, the sampling technique should be minimally invasive to the living systems; and 2) to accurately assess the mechanisms behind interactions between CNTs and contaminants, the association between the CNTs and the contaminants should not be destroyed during the sampling process. The newly established *in vivo* solid-phase microextraction (SPME) technique is an efficient tool for sampling living biology^20^,^21^,^22^,^23^. In this technique, a small amount of extracting phase dispersed on a solid support is exposed to the sample matrix for a well-defined period of time^20^. Due to its minimized morphology and slight invasiveness to a living system, the *in vivo* SPME technique can provide more accurate, more precise, and faster analytical data, and therefore, it presents a better indication of the real world in living systems^20^. In addition, the technique permits repeated temporal and longitudinal studies without having to sacrifice the individual at each sampling point, which is very important to the investigation of the thermodynamic distribution and kinetic process of the interactions between CNTs and contaminants.

In this work, we utilized the *in vivo* SPME technique to investigate the impact of CNTs on contaminant accumulation/depuration processes in living mustard plants (*Brassica juncea*) by using different organic pollutants as target analytes. In addition, the permeability and transportability of CNTs in intact mature plants were explored by employing transmission electron microscopy (TEM) and Raman spectroscopy.

## Results

### Characterization of the Carbon Nanotubes

**Figure 1:**
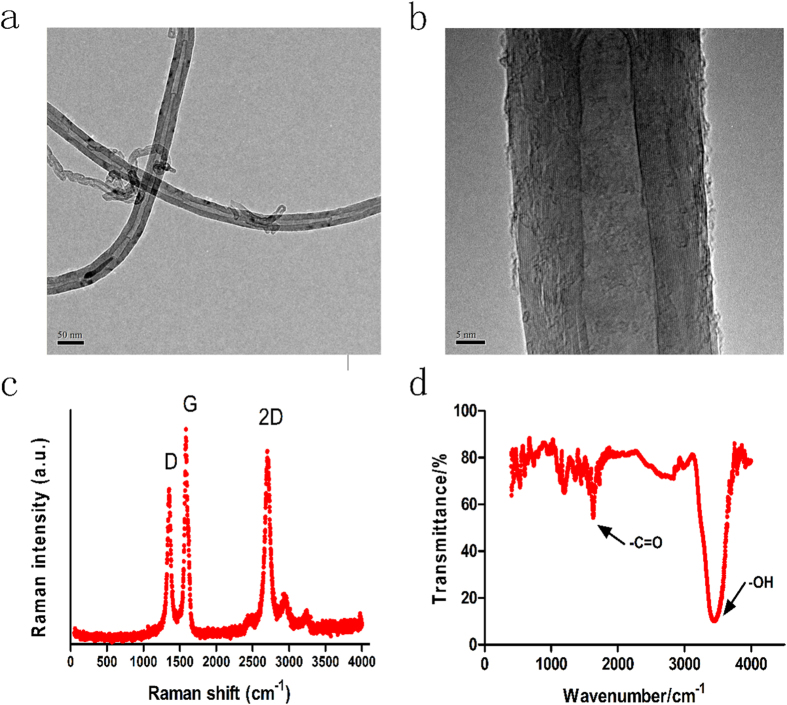
Low- (a) and high-resolution (b) TEM images of the functionalized MWCNTs and their corresponding Raman scattering spectra (c) as well as the functional group profile of the MWCNTs by FT-IR (d).

To increase the solubility of MWCNTs, the MWCNTs utilized in this study were synthesized using a modified method described by Liu *et al.*^24^. Briefly, a raw sample of MWCNTs was refluxed in nitric acid for 11 h, filtered, and washed with deionized water. The low- and high-magnification TEM images of the obtained CNTs are shown in Fig. 1A,B, which show that the inside and outside diameters of the CNTs were approximately 35 nm and 10 nm, respectively. Raman spectroscopy was utilized to analyze the crystallinity and the distribution of the diameters of the CNTs (Fig. 1C). The characteristic bands for CNTs are the D band, G band, and 2D band. The D band indicated the presence of defects and impurities in the carbon nanotubes. The G band, which generally presents between 1500 and 1600 cm^−1^, is relatively constant for CNT materials excited at different energies^25^. I_D_/I_G_ was found to be 0.80, indicating an interplanar distance of 0.342 nm between the graphite layers^26^, and I_2D_/I_G_ was 1.45, reflecting the multilayer structure. Lastly, the Fourier transform infrared spectroscopy (FT-IR) image clearly indicated the existence of carboxy groups in the modified CNTs (Fig. 1D).

### Impact on the Accumulation of Contaminants in Living Plants

Mustard plant is one of the most consumed vegetable in China and widely used as the ecological model for ecotoxicology study^27^,^28^. To comprehensively investigate the impact of the MWCNTs on the accumulation of contaminants in mustard plants, the target contaminants should cover a wide range. Here, the target contaminants included organochlorine pesticides (OCPs), organophosphorus pesticides (OPPs), pyrethroid insecticides (PYR), pharmaceuticals, and personal care products (PPCPs) (Supplementary Table 1). To test whether the MWCNTs could affect the accumulation and relative kinetic process in living plant leaves, we irrigated the mustard plants with different concentrations of MWCNTs suspended in aqueous solutions (1 and 10 μg/mL of MWCNTs with certain concentrations of the contaminants, described as MWCNTs-1 and MWCNTs-10, respectively), and a solution without MWCNTs was used for control experiments. The exposure concentration of MWCNTs was relevant with the ones applying CNTs to enhance the seed germination, root growth, and biomass of plant^13^,^14^,^15^,^16^.

**Figure 2:**
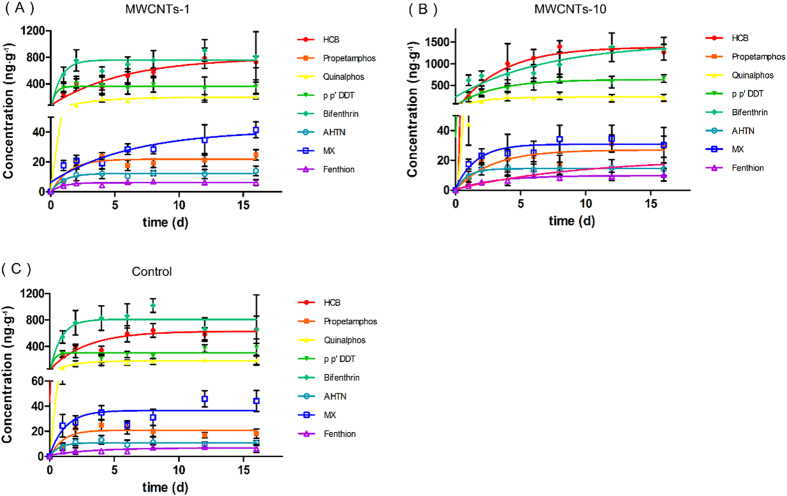
Accumulation kinetics of eight contaminants in the leaves of mustard plants irrigated with different types of exposure solutions: MWCNT-1 (A), MWCNT-10 (B), and control (C).

**Figure 3:**
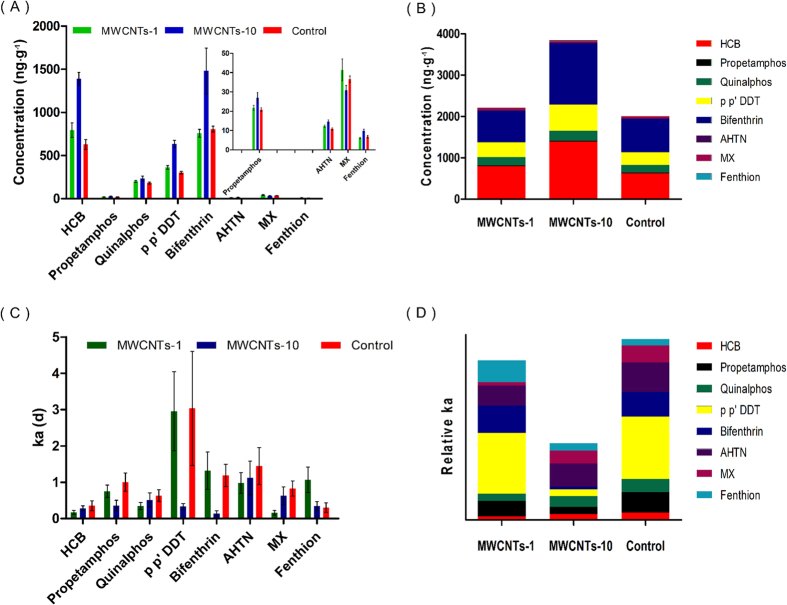
The individual concentrations (A), total concentrations (B), individual accumulation rates (C) and relative average accumulation rates (D) of eight contaminants in the leaves of mustard plants irrigated with different types of exposure media.

The accumulation kinetics of contaminants and individual concentrations of the contaminants in leaves after 16 d of exposure are displayed in Figs 2 and 3A, and the results showed that the bioaccumulations of most contaminants in the leaves increased by 10-30% (1 μg/mL) and 20-160% (10 μg/mL) after the mustard plants were irrigated with the water containing MWCNTs (Fig. 3A). In particular, the concentrations of p-p’ DDT and HCB (in the MWCNTs-10 group) increased by 110% and 121%, respectively, compared with the control group. To clearly reflect the impact, the total concentrations of the contaminants were compared (Fig. 3B). The results obviously indicated that the existence of the MWCNTs enhanced the accumulation of the contaminants in the mustard plants, and the enhanced impact of the contaminants was dependent on the concentration of MWCNTs.

Furthermore, we analyzed the impact of the MWCNTs on the accumulation rate constants (*k*_*a*_) of the contaminants. Figure 3C,D show that the existence of the MWCNTs generally decreased the *k*_*a*_ values of the contaminants in mustard plants, which is contradictory to the impact of the accumulation, which resulted in enhanced values. For example, the accumulation rate constants of bifenthrin (MWCNTs-10) and MX (MWCNTs-1) decreased by 88% and 81%, respectively. However, it is worth noting that the times for the contaminants to reach their plateaus in leaves were prolonged when the plants were exposed to the groups containing MWCNTs (Fig. 2), which caused the contaminant accumulations to be enhanced compared with those of the control group (Fig. 3A,B).

### Impact on the Depuration of Living Plants by Contaminants

**Figure 4:**
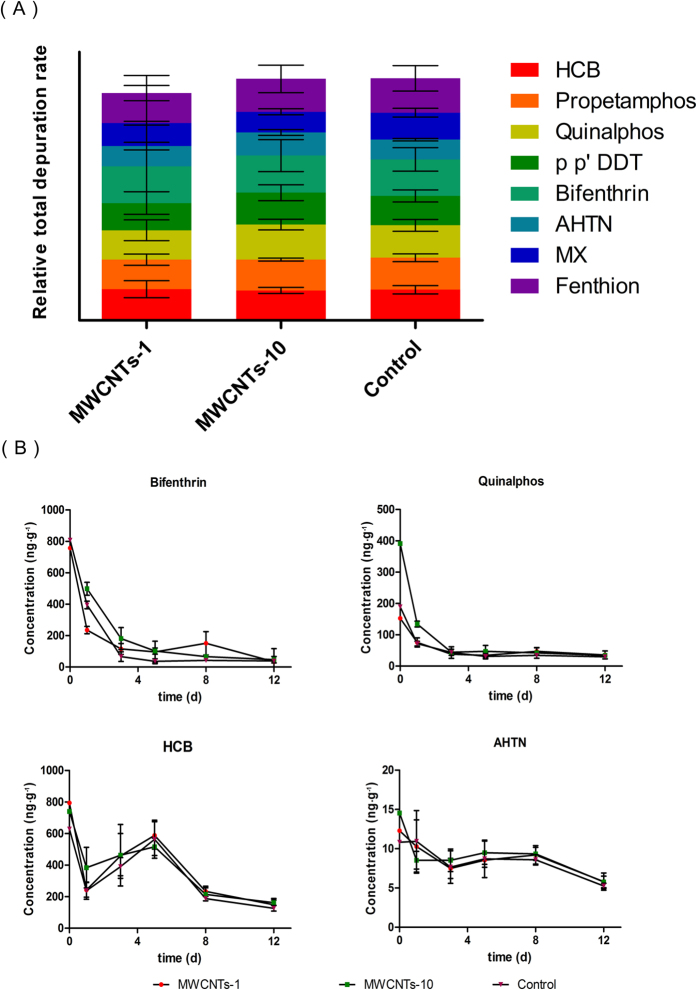
Total relative depuration rates in the leaves of mustard plants after irrigation with clear water for 12 d (A) and the corresponding depuration kinetics of bifenthrin, quinalphos, HCB, and AHTN in the leaves (B).

We further investigated the impact of the MWCNTs on the depuration capacities of the plants for the contaminants. To achieve this goal, after tracing the contaminant accumulation, the same plants were merely irrigated with clear water, and the concentrations of the contaminants in the leaves were traced for 12 d (Supplementary Fig. 1). In general, the depuration capacities of the plants for the contaminants were not significantly changed after suffering 16 d of exposure to MWCNTs (Fig. 4A). The anti-biodegradable pollutants, such as OCPs (HCB and p-p’ DDT) and synthetic musks (AHTN, MX), still displayed slow depuration rates. For example, the depuration rate of HCB was less than 50% after 5 d, and the decrease in the concentration of AHTN was not significant after 12 d (Fig. 4B). On the other hand, the biodegradable pollutants, such as OPPs (propetamphos, quinalphos, and fenthion) and PYR (bifenthrin), showed excellent decreasing rates with and without exposure to MWCNTs. For example, the depuration rates of quinalphos and bifenthrin were more than 70% and 88% after 5 d (Fig. 4B).

### Permeability and Transportability of the MWCNTs

The Permeability of CNTs in the seeds and seedlings was confirmed previously^10^. However, the relative studies regarding to the mature plants, were remain, because of the existence of stronger cell wall in the mature stage. To understand the fates of the MWCNTs during the exposure process, we attempted to capture traces of MWCNTs inside the mature plants. Raman spectroscopy is a technique that can give accurate information about the presence of graphite-based materials, such as CNTs, inside a biological system^10^. The strong and specific Raman scattering signal of the G band of individual CNTs and their clusters, which is present between 1500 and 1605 cm^−1^, allowed this technique to accurately detect the CNTs among the biological tissues of the plant. Due to the non-destructive *in vivo* sampling technique, the mustard plants were able to be used for Raman spectroscopy.

**Figure 5:**
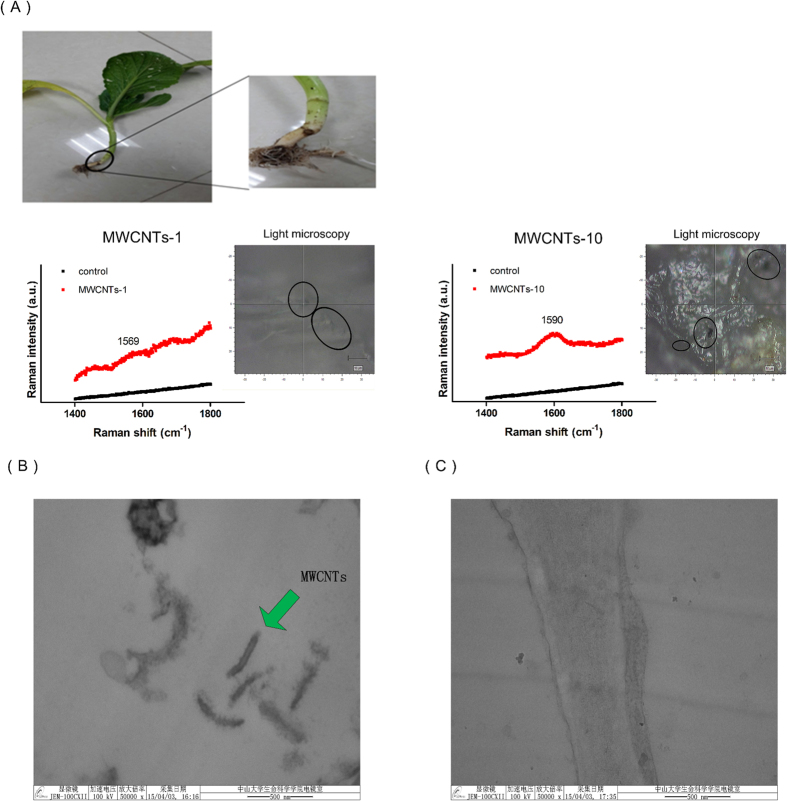
Raman scattering detection of aggregates of MWCNTs in mustard roots (A). The presence of a characteristic peak can be observed in several spots along the surfaces of slices from the CNT-exposed plants. The peak corresponds to the G band of the MWCNTs, and it was not detected in the control samples. The high-magnification TEM images of a leaf of a mustard plant exposed to a MWCNT group (**B**) and a control group (**C**). The tubular structure was only captured in the leaf from the MWCNT group.

After the accumulation tracing experiment was finished, several mustard plants were removed from the soil and washed with tap water. Their anatomical leaves and roots were cut into three slices, and the freshly exposed surfaces were analyzed by Raman spectroscopy. For the roots, Raman signals of the G band (1569, 1590 cm^−1^) of the MWCNTs were found in slices of root exposed to two different concentrations of MWCNTs, respectively, and the intensities of the signals reflected the different degrees of aggregation (Fig. 5A). It is worthwhile to note that the signal was detected in all three slices of the roots exposed to MWCNTs-10, while the signal was only found in one of the slices exposed to MWCNTs-1, and that signal was weak. The images from light microscopy provided more direct evidence to support the existence of the MWCNTs inside the roots of the plants, as shown in Fig. 5A. Moreover, the MWCNTs inside the roots could aggregate to form particles with diameters of more than 1 μm (Supplementary Fig. 2A). However, no signal was detected in all of the slices without MWCNTs exposure, even though the acquisition time was relatively long (Supplementary Fig. 2B). The experimental results demonstrated that the MWCNTs could penetrate the cell walls of mature mustard plants.

The transportability of MWCNTs to the upper organs (e.g., the edible parts such as the leaves and fruit) within the intact mature plant still remained to be investigated. In this study, no dark spots were found in all of the leaf slices viewed under a light microscope, and due to the strong fluorescence interference of leaves, the corresponding G band of the MWCNTs was not observed under Raman spectroscopy. To confirm whether the MWCNTs existed in the leaves, another TEM experiment was conducted. The tubular structures of the MWCNTs were captured in sliced leaves under high-magnification TEM (Fig. 5B), and the dimensions of the nanotubes were consistent with those in Fig. 1B. Meanwhile, no similar structure was observed in any of the leaves of the control plants (Fig. 5C and Supplementary Fig. 3). This finding illustrated that the MWCNTs could translocate from the roots to the upper organs within intact mature plants under the force of the transpiration steam.

### The Mechanism of CNT-contaminant Interactions

It has been reported that water soluble substances absorbed on organic matter, which was assimilated by the plant, could be released inside the plant and then migrate to the other compartments under the force of the transpiration stream^29^. The investigation indicated that the contaminants adsorbed on the MWCNTs may also be bio-available and may affect the distribution and transportation of the contaminants in the plants.

To reveal the mechanism influencing contaminants enrichment in plant, firstly, it need to be considered if the relative enrichment effect was “nano-related”. Herein, carbon fiber (tex number 200, filament diameter 0.007 mm), was selected as a micro-sized carbon material for control test. As shown in Supplementary Fig. 4, due to the adsorption of carbon fiber toward organic matters and the cellular impermeability of micro-sized material (Supplementary Fig. 5)^30^, the concentrations of the contaminants accumulated in leaves of mustard plants were lower than the control group only contained tap water (16 d exposure). The results indicated the enrichment effect of contaminants was “nano-related”.

**Figure 6:**
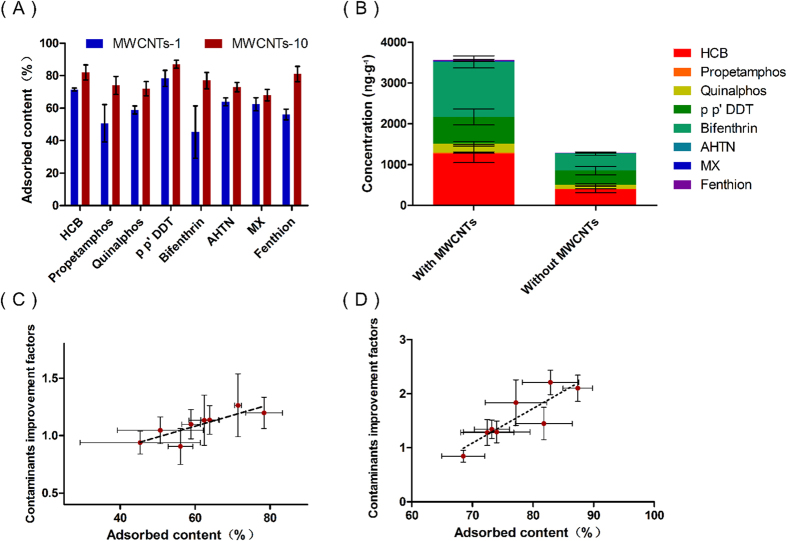
The adsorbed contents of contaminants in two types of exposure solutions: MWCNTs-1 and MWCNTs-10. (A) The total concentrations of contaminants in the leaves of a mustard plant irrigated with spiked solutions with or without MWCNTs for 16 d (**B**), with the spiked solutions containing consistent free concentrations of the contaminants. The linear relationships between the adsorbed contaminant contents on MWCNTs and the contaminant improvement factors for the MWCNTs-1 group (**C**) and the MWCNTs-10 group (D). The linear correlation coefficients (R^2^) were 0.69 and 0.76, respectively.

To study the specific mechanism toward MWCNTs, adsorption contents of contaminants on MWCNTs were investigated. Due to the multilayer microtubular structure and hydrophobic nature of CNTs, the adsorbed contaminant contents on MWCNTs were generally higher than 50% (Fig. 6A and Supplementary Fig. 6). Generally, free contaminants are considered to be bio-available. To confirm if the contaminants adsorbed by the MWCNTs were bio-available, the mustard plants were irrigated with spiked solutions with or without MWCNTs, which contained equal free concentrations of the contaminants (the free concentrations of the contaminants are listed in Supplementary Table 2). As shown in Fig. 6B, the total concentration of the contaminants in the leaves of the plants irrigated with the solution of MWCNTs was significantly higher than that in the leaves of the control plants, which revealed the bio-availability of the contaminants adsorbed by the MWCNTs. In addition, the improvement factors, which were defined as the ratio of the cumulative concentrations of the contaminants between the MWCNTs groups (MWCNTs-1 and MWCNTs-10) and the control group, were positively correlated with the adsorbed contents of the contaminants (Fig. 6C,D). Moreover, the decreases in the *k*_*a*_ values were negatively correlated with the adsorbed contents of the contaminants (Supplementary Fig. 7).

These findings demonstrated the following: 1) most of the contaminants were adsorbed by the MWCNTs and therefore deceased the *k*_*a*_ values of the contaminants; 2) the chronic release of the adsorbed contaminants in plants led to a prolonged time for the contaminants in the leaves to reach accumulation plateaus (Fig. 2), but the concentrations of the contaminants in the leaves will be greater in the presence of MWCNTs. This phenomenon was “nano-related”.

## Discussion

In this study, for the first time, we demonstrated that MWCNTs can act as contaminant carriers, influencing the accumulation of contaminants in crops, and the relative effects varied depending on the adsorbed content of contaminants on the MWCNTs. To date, it was widely believed that specific types of nanoparticles in low doses were not harmful to plants^10^,^15^,^16^. However, as water and soil pollution have become global environmental issues that threaten plant growth, herein, we proved that MWCNTs in low doses could enhance contaminant accumulation in crops.

In addition, we provided the first evidence that CNTs could penetrate into the roots of intact mature plants and then be transported to the upper organs. Additionally, the MWCNT-adsorbed compounds could be released inside the plant, which may provide a route to effectively deliver drugs or genetic materials to specific sites of intact plants. However, some large black spots observed under light microscopy indicated that the MWCNTs aggregated within the roots, which might cause negative effects, such as inducing potential nanotoxicity, inhibiting nutrient transport, and affecting plant growth (e.g., leaf color to amaranthine was observed in this study, Supplementary Fig. 8). For human safety and health, more investigations on nano-agriculture need to be conducted to avoid possible increased exposure to contaminants and nanoparticles, especially in the roots of edible plants.

## Methods

### Exposure Experiment

The plant growth conditions and the preparation of the relative exposure solutions are provided in the Supplementary Text. For accumulation studies, each group (MWCNTs-1, MWCNTs-10 and control group) contained eight mustard plants. The supply of MWCNTs to pots was carried out through regular watering with tap water, which was referenced to 15 (the details of irrigation method were provided in Supplementary Text). After 16 d of tracing, three plants were randomly removed for the analysis of the CNTs, while the others were merely irrigated with tap water regularly for the depuration studies.

### *In vivo* Sampling

The PDMS fibers used for *in vivo* sampling were prepared in our lab^31^, and the preparation process is supplied in Supplementary Fig. 9. The *in vivo* sampling rates, which were used for qualification, and the details of the *in vivo* sampling process are provided in the Supplementary Text, Table 3, and Fig. 10.

### Detection of the Contaminants

For contaminant detection, analysis of the contaminants was performed on an Agilent 6890N gas chromatograph equipped with a MSD 5975 mass spectrometer and electron-impact ionization (details are provided in the Supplementary Text).

### Raman Spectroscopic Analysis

For detection of the MWCNTs, fresh slices of root and leaf were immobilized on glass slides and then analyzed by laser-micro-Raman spectroscopy at room temperature. The Raman spectroscopy conditions are provided in the Supplementary Text.

### Transmission Electron Microscopy

The separated leaf tissue (<1 cm^3^) was overlaid with a fixing solution containing 5% glutaraldehyde and 4% paraformaldehyde, and then it was exhaustively evaporated with a vacuum pump. The glutaraldehyde was removed after 2 d, and 2% osmium tetroxide was added to the fixation followed by rinsing with 0.1 M PBS buffer (pH = 7.2). After dehydration (rinsing with gradient ethanol five times, 20 min/time), 0.25 mL of epoxy was added and refreshed three times (2 h/time), and then the tissue stood for 12 h preceding the embedding of the tissue into Spurr’s resin. Thin sections were cut using an ultramicrotome equipped with a diamond knife (Leica EM UC7). Sections were mounted on copper grids, and the sections were examined by transmission electron microscopy (JEM-100CXП) under 100 kV, 50000×.

## Additional Information

**How to cite this article**: Chen, G. *et al.* Carbon Nanotubes Act as Contaminant Carriers and Translocate within Plants. *Sci. Rep.* **5**, 15682; doi: 10.1038/srep15682 (2015).

## Supplementary Material

Supplementary Information
