# Supplementary material for: Carbon Nanotubes Act as Contaminant Carriers and Translocate within Plants
Source: Sci Rep. 2015 Oct 26;5:15682. doi: 10.1038/srep15682 (PMC4620501; doi:10.1038/srep15682)
Supplement: Supplementary Information [file srep15682-s1.pdf]

Supplementary Information

Carbon Nanotubes Act as Contaminant Carriers and Translocate within Plants

Guosheng Chen[a], Junlang Qiu[a], Yan Liu[a], Ruifen Jiang[a], Siying Cai[a], Yuan Liu[b], Fang Zhu[a*], Feng Zeng[a], Tiangang Luan[a], Gangfeng Ouyang[a*]

[a]*MOE Key Laboratory of Aquatic Product Safety/KLGHEI of Environment and Energy Chemistry, School of Chemistry and Chemical Engineering, Sun Yat-sen University, Guangzhou 510275, China*

[b]*Department of Food Science and Technology, College of Food Science and Technology, Shanghai Ocean University, Shanghai 201306, China.*

[*]Corresponding author. Tel. & Fax: +86-20-84110845

E-mail: ceszhuf@mail.sysu.edu.cn (F. Zhu); cesoygf@mail.sysu.edu.cn (G. Ouyang).

Page: 18

Text: 1

Figure: 9

Table: 4

Supplementary Text

Plant Growth Conditions and Preparation of the Exposure Mediums. The mustard plants used in this study were cultivated from seeding in a plant incubator (Conviron A1000, Canada) under 14 h light (25 ℃) and 10 h dark (23 ℃)，40% humidity (the soil used for plant growth was characterized in Supplementary Table 4, and each pot contained equivalent soil). The mature plants (three-month-old) were used to exposure experiment. MWCNTs suspensions were prepared using an ultrasonic processor (Sonics 04711-35, USA) operated at 500 W. Probe sonication was conducted by using a 20 s on/40 s off pulse sequence with a 13 mm diameter probe tip. Three types of exposure mediums were used for experiment: 200 μg/L contaminants spiked water (HCB and p-p’ DDT were 50 μg/L, the spiked water described as CW), CW supplemented with 1 μg/mL MWCNTs suspensions (MWCNTs-1) and CW supplemented with 10 μg/mL MWCNTs suspensions (MWCNTs-10).

Irrigation Method. All of the plants were watered once at day with tap water. The plants from control group were watered with contaminants spiked water (without MWCNTs added) additionally twice a week, and the plants from and MWCNT groups were additionally watered with suspensions containing 1 μg/mL of MWCNTs (group MWCNTs-1) or suspensions containing 10 μg/mL of MWCNTs (group MWCNTs-10) twice a week. To achieve this task, the contaminants spiked water or MWCNTs suspensions (50 mL for each used concentration) was added inside of soil into each experimental pot

***In vivo* Sampling.** In this study, three leaves of each plant were conducted to sampling.

The *in vivo* sampling process was as follow: The petiole of mustard plants was pierced with a 26 gauge hypodermic needle to a depth of approximately 1.4 cm. Subsequently, the needle was removed, and the custom-made PDMS fiber was deployed in the punched hole (Supplementary Fig. 10). Two parallel samplings in both sides of each petiole were conducted at each sampling point for mutual reference. After 20 min extraction duration, the fiber was removed, rinsed with deionized water and dried with a Kimwipe tissue, and then assembled to a recycled SPME fiber assembly for being directly introduced to GC-MS for analysis.

Determination of *in vivo* Sampling Rates. In the Sample Rate-SPME model (1), it assumes that the sampling rate R_s remains constant when the extracted amount is less than 50% of the equilibrium amount. The concentration of target analyte in the sample matrixes (C_0) can be expressed with the following equation:

$$C_0 = n/(R_s\ t) \qquad (1)$$

where n is the amount of the extracted analyte in fiber, and t is the sampling time. It assumes that the variance of R_s between individuals is acceptable.

Eq. 1 showed that if the parameters of fiber extraction amount n, and initial sample concentration C_0 were measured, the *in vivo* sampling rate R_s could be obtained in a certain extraction duration. Six mustard plants were irrigated with 50 mL 1000 $\mu g \cdot L^{-1}$ spiked water (HCB and p-p' DDT were 250 $\mu g \cdot L^{-1}$) once a day. After 3 d exposure, the leaves of mustard plants was extracted by *in vivo* SPME and quantified by GC-MS. Since the non-exhaustive extraction nature of *in vivo* SPME, the concentrations of target analyte in the sample matrixes were insignificantly changed after extraction, so the

initial sample concentration C_0 could be measured using liquid extraction after fiber extraction (as seen below). The *in vivo* sampling rates for contaminants in leaves of mustard plants were displayed in Supplementary Table 2 and used to quantify in the current research.

Liquid Extraction. Liquid extraction methods were used to determine the concentrations of contaminants in leaves of mustard plants. After *in vivo* sampling for 20 min, 2 g tissues of leaves were removed and homogenized with a blender. The homogenized tissues were transferred into a conical flask of 50 mL, 15 mL of MTBE were added. The mixture was ultrasonic extracted for 0.5 h, and subsequently ultrasonic extracted another 0.5 h after 15 mL of acetonitrile was added. After filtration, 4 g sodium sulfate was added to the upper solution to remove the remaining water. The supernatant was decantated into evaporation pan and evaporated to 1 mL in water bath (60 ℃) and then transferred to a graduated test tube. Feed the volume to 2 mL with acetone.

Adsorbed Contents of the Contaminants on CNTs. To study the adsorbed capacity of contaminants on MWCNTs, we used equilibrium SPME (immersion model) with custom-made PDMS fiber to trace the free concentrations of contaminants in the MWCNTs-contaminants suspension system for 7 d. The extraction temperature was 25 ℃ and the stirring rate was set at 1000 rpm, extraction time was optimized and 80 min was selected (Supplementary Fig. 11).

GC-MS. For contaminants detection, the analysis of contaminants was performed on an Agilent 6890N gas chromatograph equipped with a MSD 5975 mass spectrometer

and electron-impact ionization (EI). A split/splitless-type injector was used for sample introduction. Chromatographic separation was carried out with a HP-5MS capillary column (30 m × 250 μm × 0.25 μm, Agilent Technology, CA, USA). The inlet temperature was 250 ℃, and the oven temperature programs were as follows: The initial oven temperature was 80 ℃ (held for 0. 5 min), ramped at 30 ℃/min up to 250 ℃ (held for 5 min), and ramped at 30 ℃/min up to 280 ℃ (held for 5 min). Helium was used as carrier gas at a constant flow rate of 1.2 mL/min. The MSD was operated in the electron impact ion (EI) mode with a source temperature of 230 ℃. The electron energy was 70 eV and the filament current 200 A.

Raman Spectroscopy Analysis. For MWCNTs detection, the freshly slices of root and leaf were immobilized on the glass slides and then analyzed by Laser-Micro-Raman spectroscopy at room temperature. Raman scattering spectra were recorded using a Renishaw in Via equipped with a charge-couple-detector, and a spectrometer with grating of 600 lines/mm. a Ar^+ laser (514 nm) was used as the excitation source. The laser beam intensity measured at the sample was 20mW, and Raman shifts were calibrated with a silicon wafer at a peak of 521 cm^{-1}.

References

1. Ouyang, G. *et al*. Sampling-Rate Calibration for Rapid and Nonlethal Monitoring of Organic Contaminants in Fish Muscle by Solid-Phase Microextraction. *Environ. Sc. Technol.* **45**, 7792–7798 (2011).

Supplementary Figure

Suppl Fig. 1

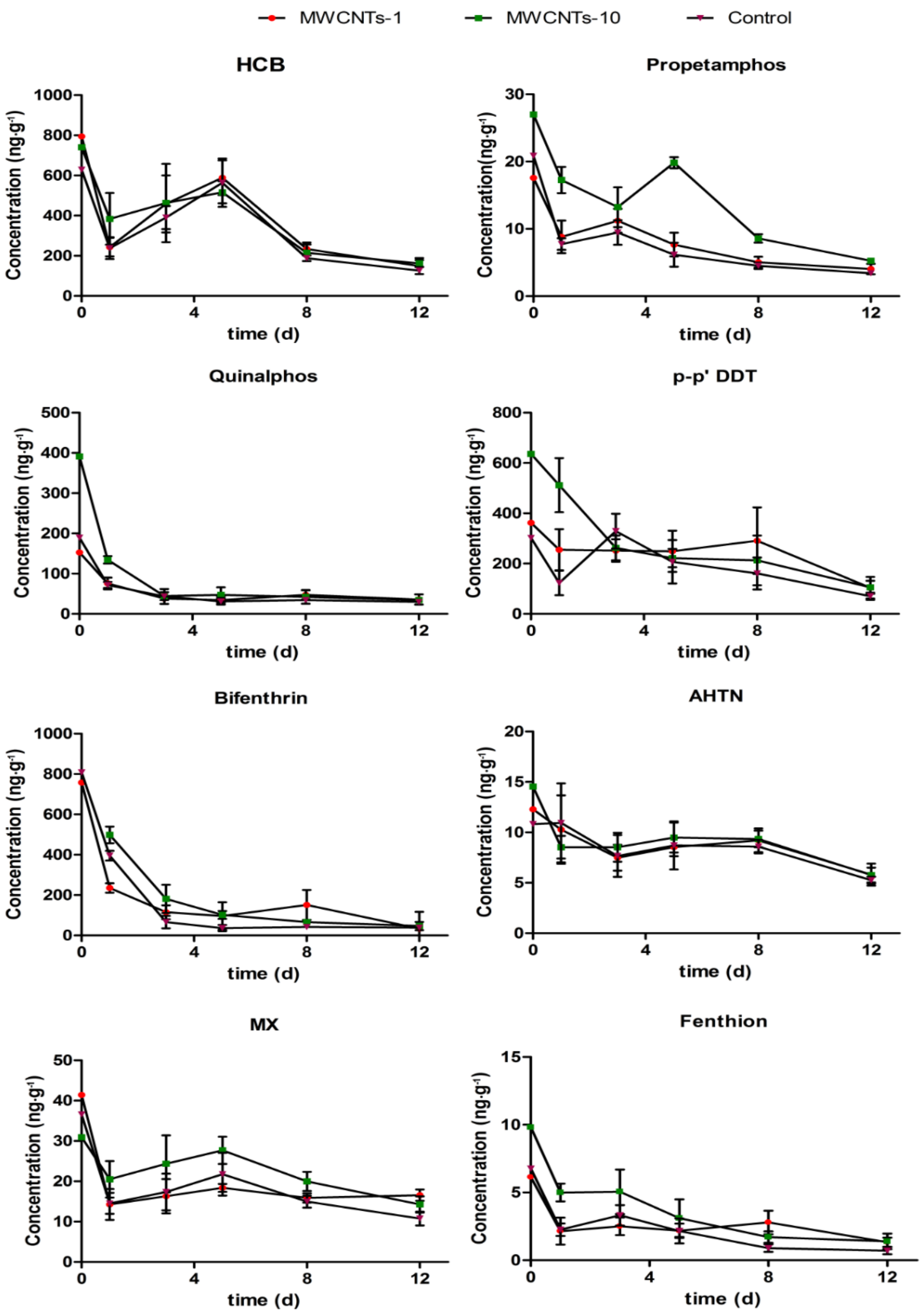

Supplementary Fig. 1. Depuration kinetics of eight contaminants in the leaves of mustard plants, which have been irrigated with three types of exposure solutions (MWCNTs-1, MWCNTs-10 and control group) for 16 d. Error bar presented the standard deviation.

Suppl Fig. 2

(A)

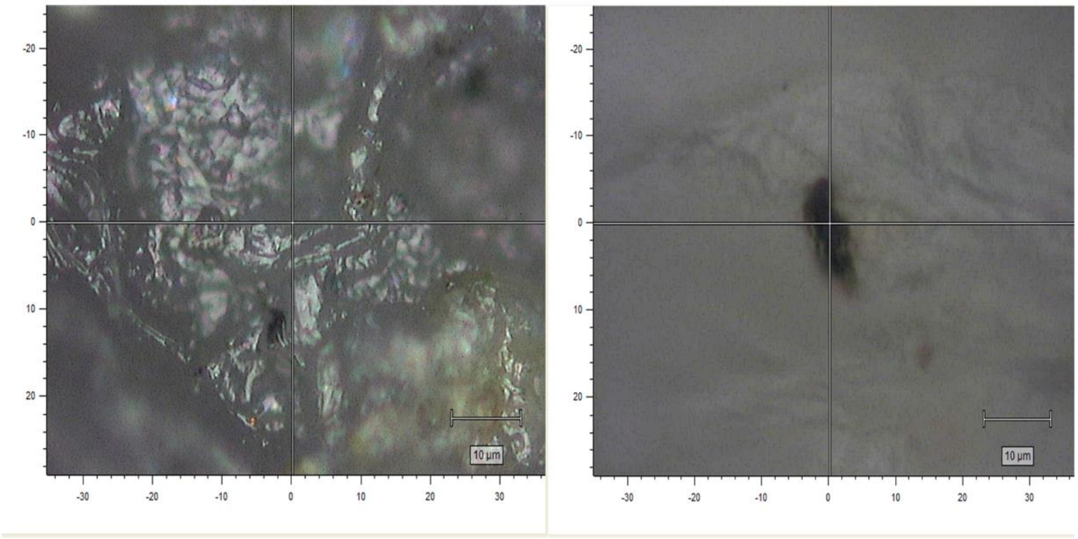

(B)

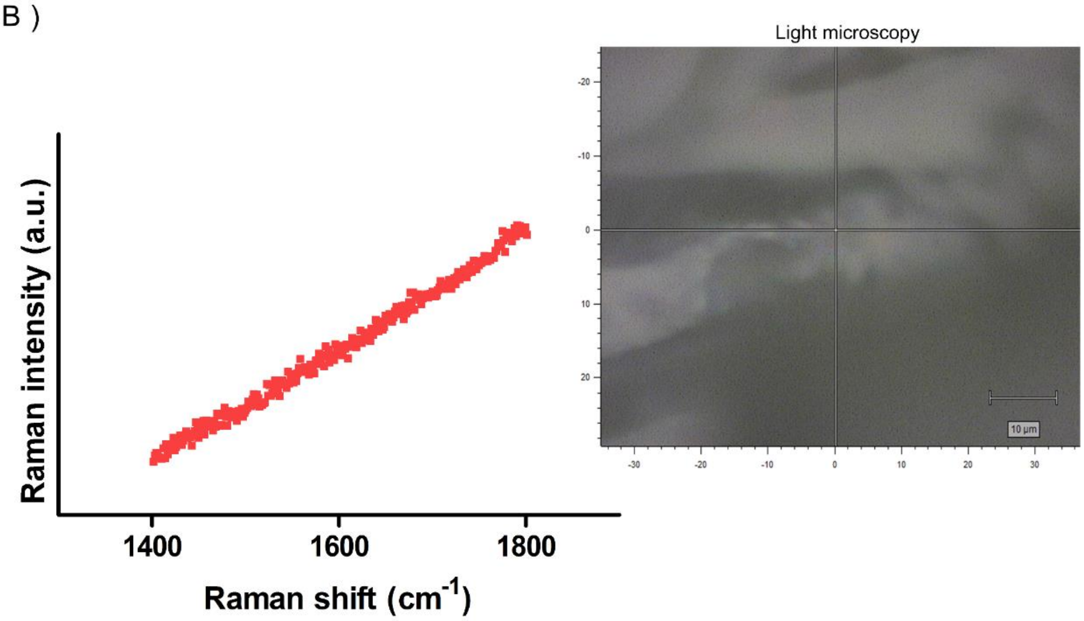

Supplementary Fig. 2. The dark spots (MWCNTs aggregates), with the diameter > 1 μm, were observed in the roots of mustard plants exposed to MWCNTs solution, by light microscopy (A). Raman-scattering signals were not observed in the root of mustard plant without MWCNTs exposure, meanwhile, no dark spot was found along the surface of the root slices (B).

Suppl Fig. 3

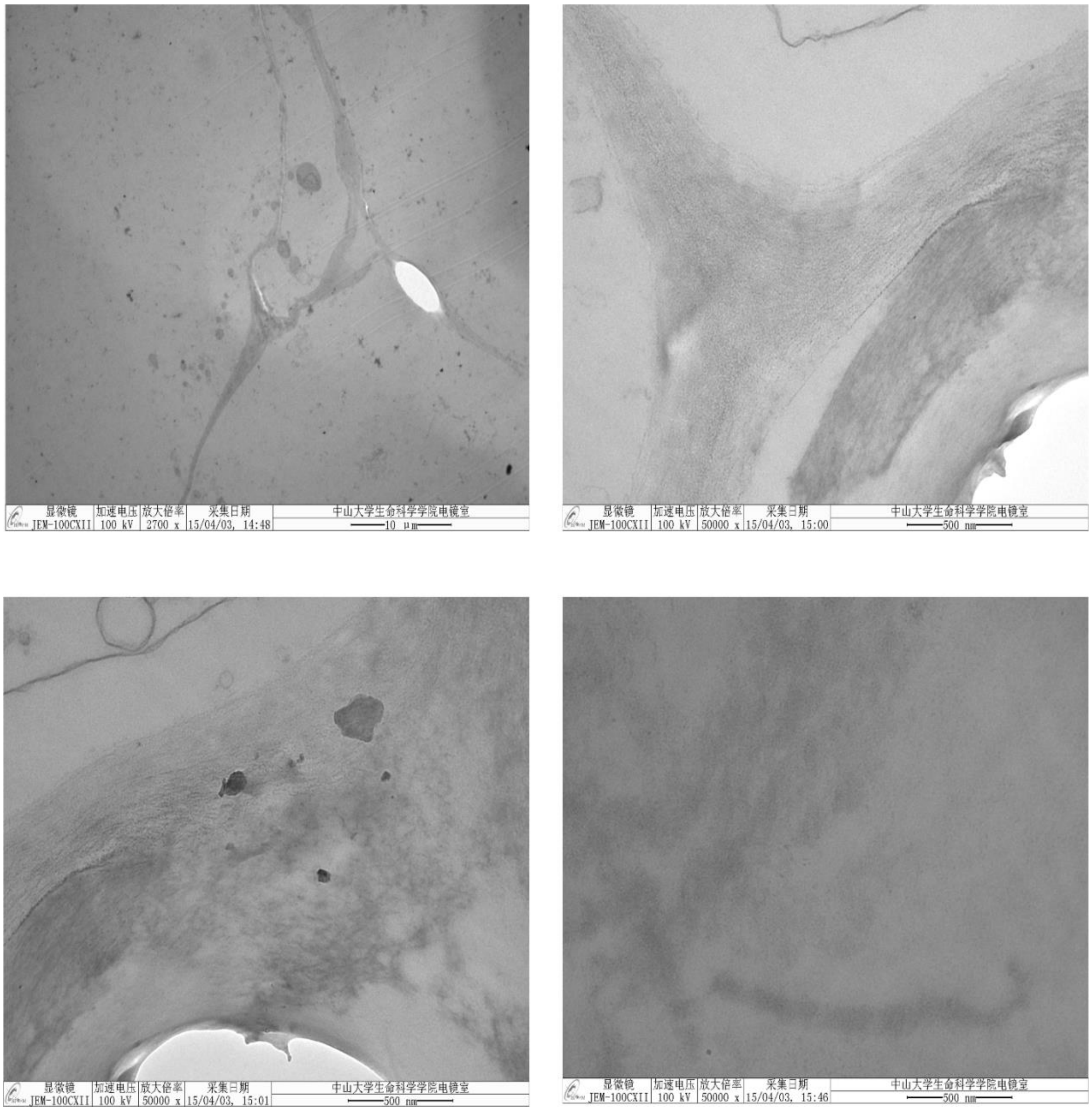

Supplementary Fig. 3. The high-magnification TEM of other sliced leaves from control group. It clearly reflected that no tubular structure was discovered in any sliced leaves.

Suppl Fig. 4

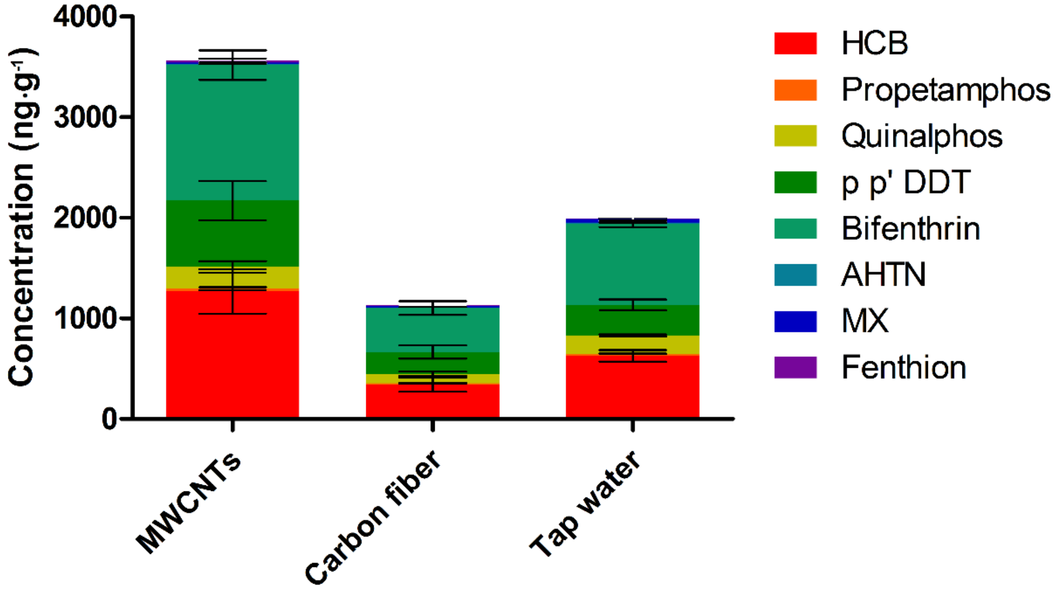

Supplementary Fig. 4. The concentrations of contaminants in the leaves of a mustard plant irrigated with spiked solutions with MWCNTs (10 μg/mL) and carbon fiber (10 μg/mL) for 16 d, and the spiked tap water was used as a control. The spiked solutions of the three groups was containing consistent total concentrations of the contaminants.

Suppl Fig. 5

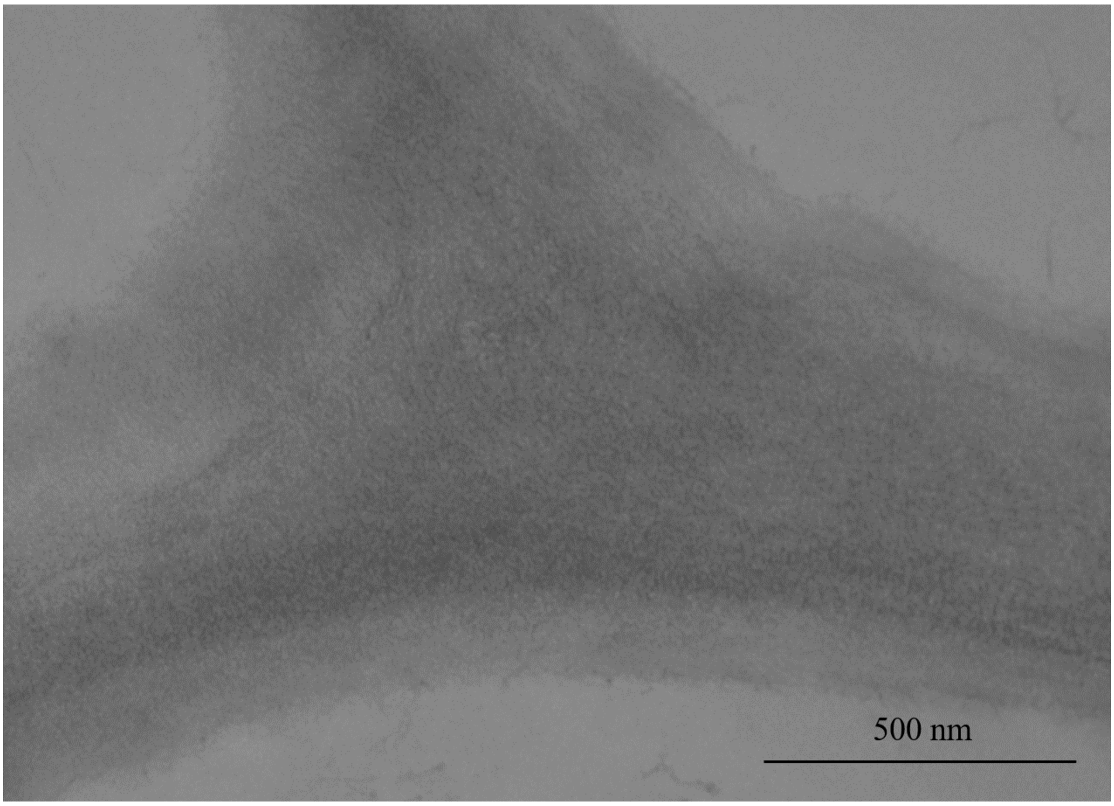

Supplementary Fig. 5. No fiber was observed in the leaf of plant exposed to carbon fiber solution.

Suppl Fig. 6

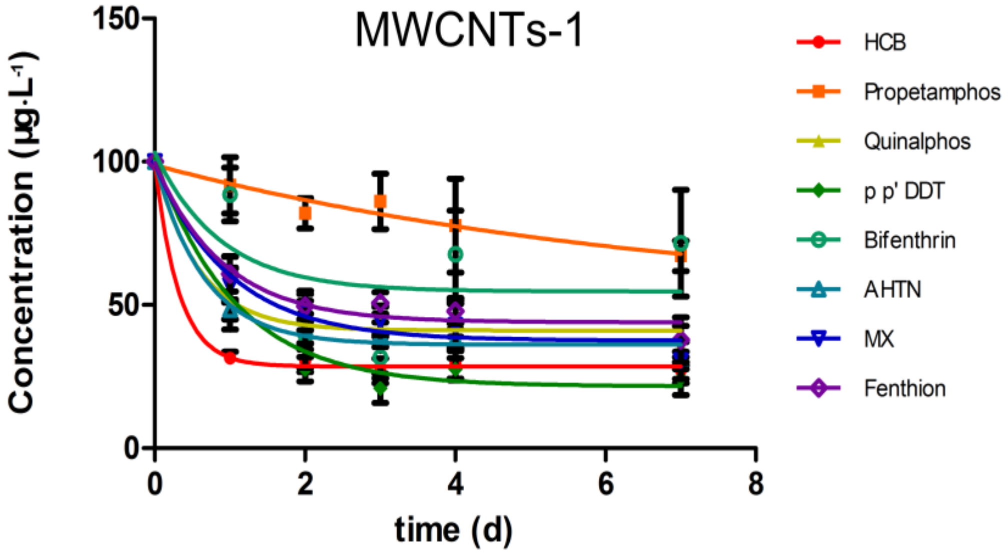

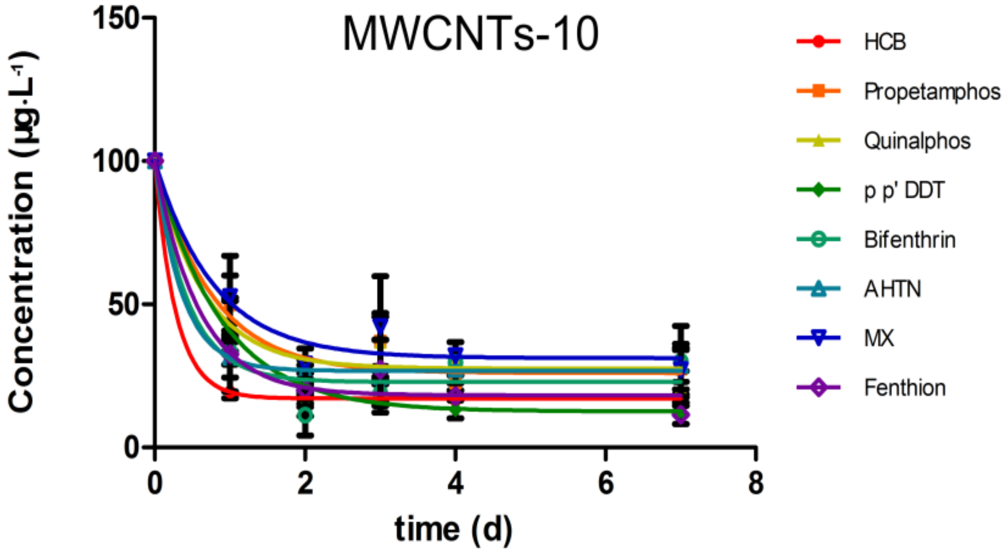

Supplementary Fig. 6. Tracing the free concentrations of contaminants in two types of MWCNTs-contaminants suspension: MWCNTs-1 and MWCNTs-10. Error bar presented the standard deviation

Suppl Fig. 7

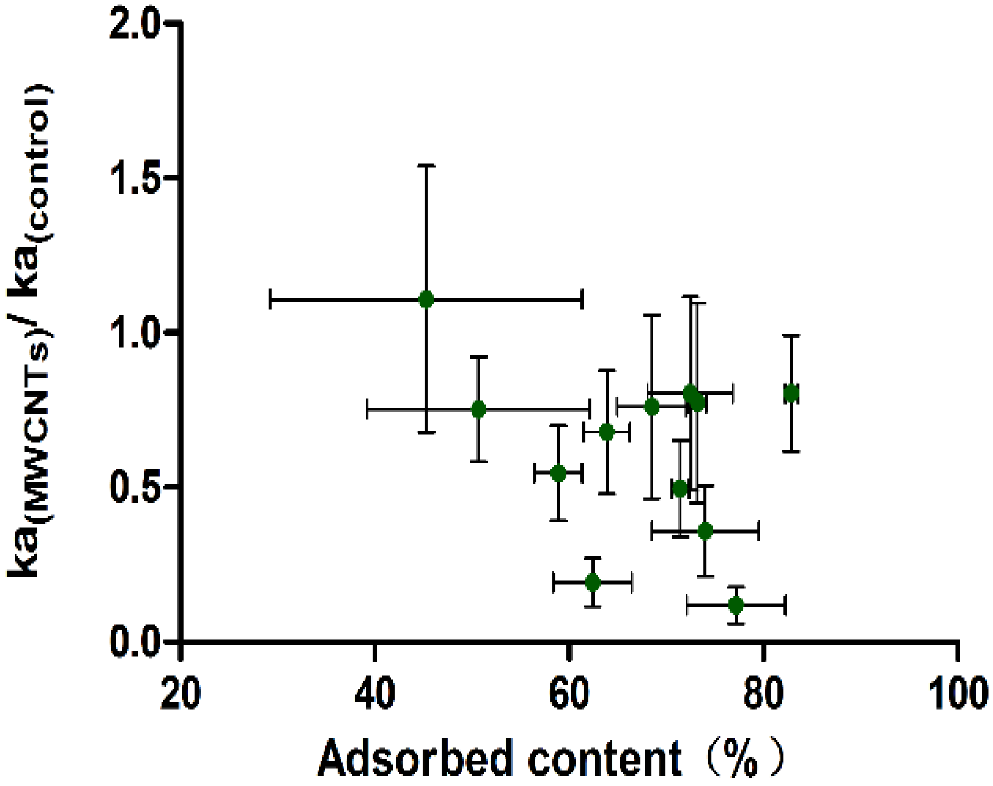

Supplementary Fig. 7. The relationship between adsorbed contents of contaminants on MWCNTs and the corresponding ratios of $k_{a\ (MWCNTs)}$ and $k_{a\ (Control)}$. Here, p-p' DDT was removed due to the high SD of the value of k_a. It showed that the inhibited impact on the value of k_a became stronger when the adsorbed content of contaminant on MWCNTs was increasing.

Suppl Fig. 8

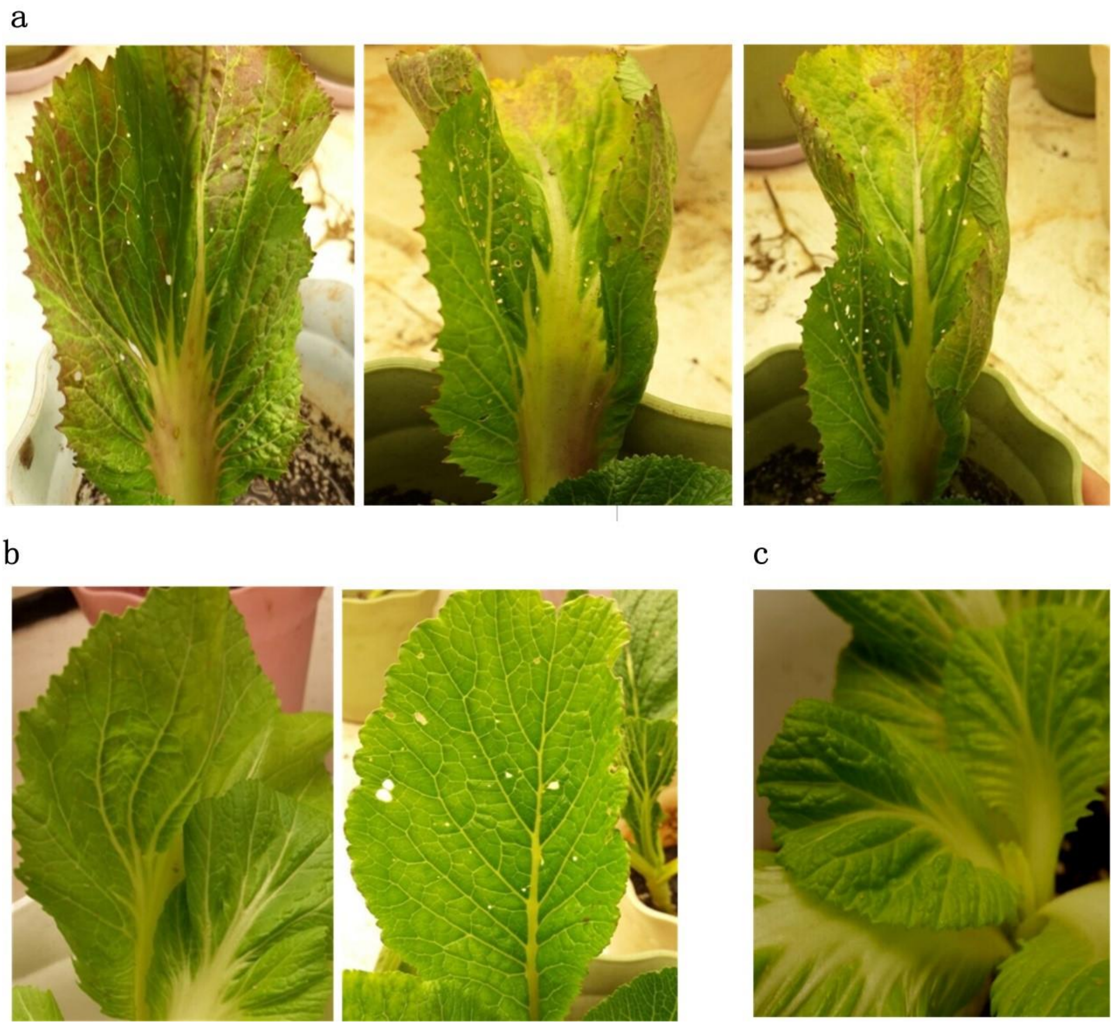

Supplementary Fig. 8. Impacts of the MWCNTs on the morphology of mustard plants： a) irrigated with MWCNTs-10 solution, b) irrigated with control solution, c) irrigated with tap water. All the plants was grown in the identical condition. The leaf colored to amaranthine was partly observed in (a), while the corresponding change was not found in (b) and (c).

Suppl Fig. 9

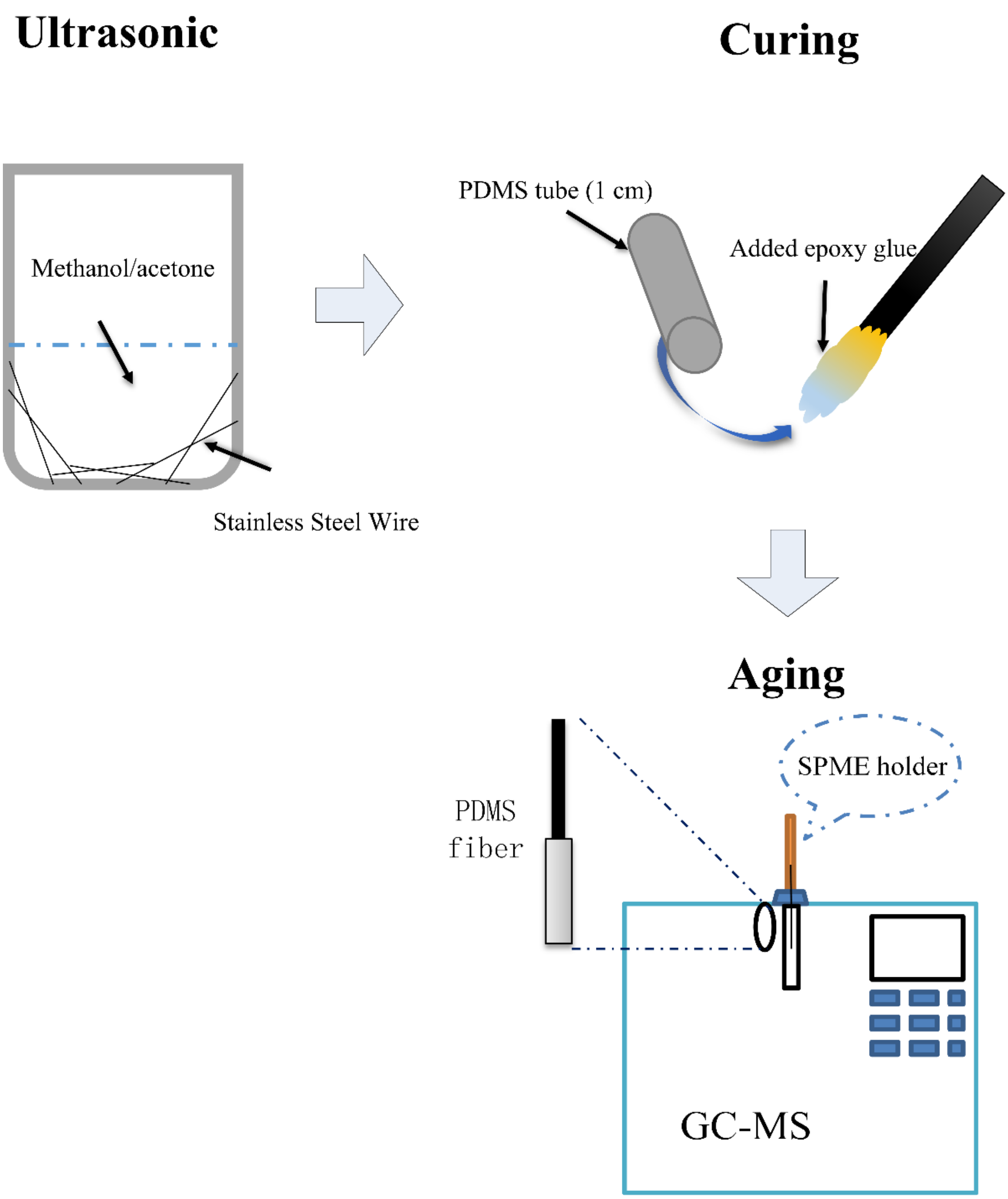

Supplementary Fig. 9. The preparation schematic diagram of custom-made PDMS fiber.

Suppl Fig. 10

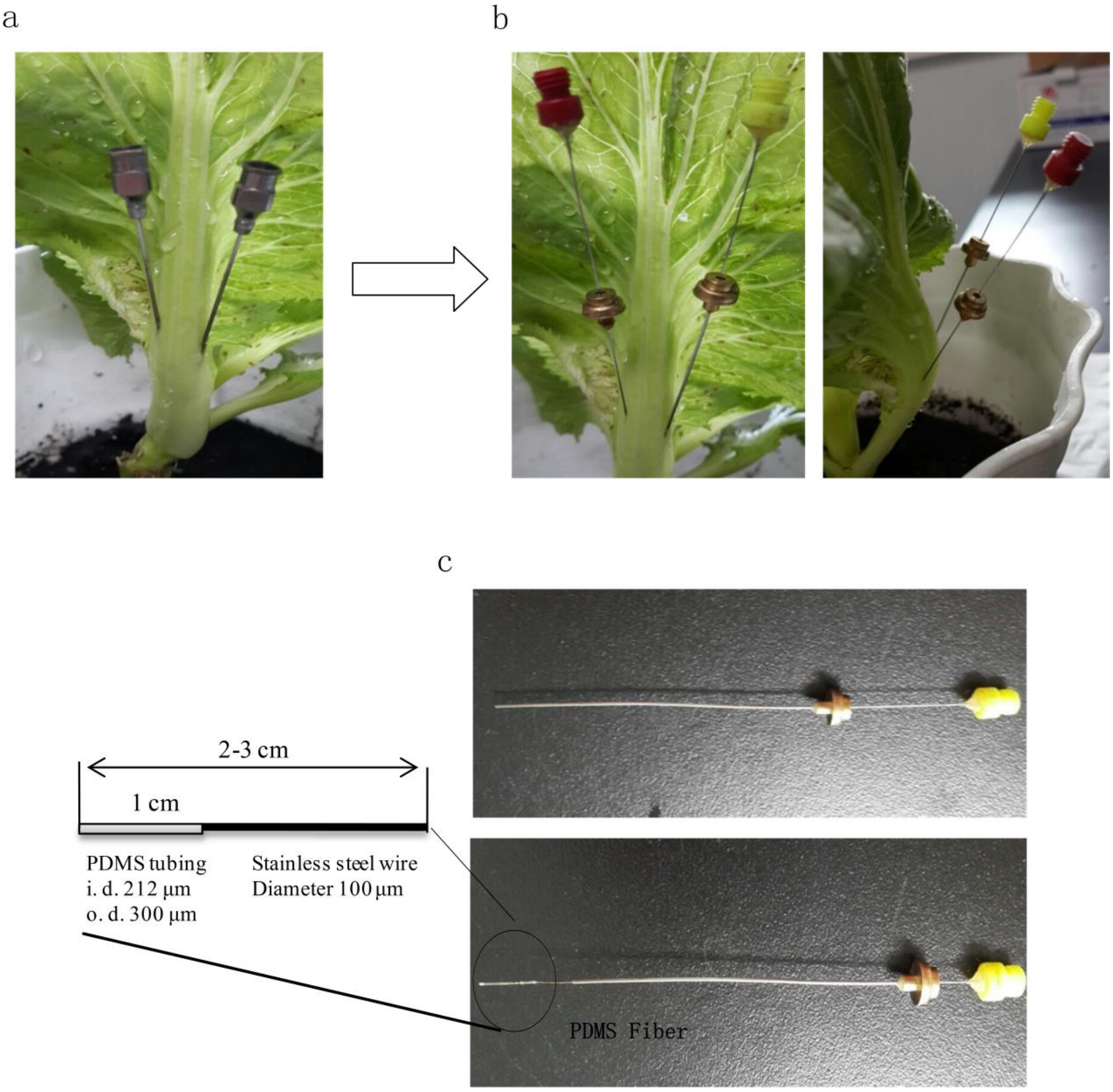

Supplementary Fig. 10. The schematic diagram of *in vivo* SPME in mustard plant leaf. a) The petiole of mustard plants was pierced with a 26 gauge hypodermic needle to a depth of approximately 1.4 cm, b) two parallel samplings in both sides of the petiole were conducted at each sampling point, c) the custom-made 44 µm PDMS fiber.

Suppl Fig. 11

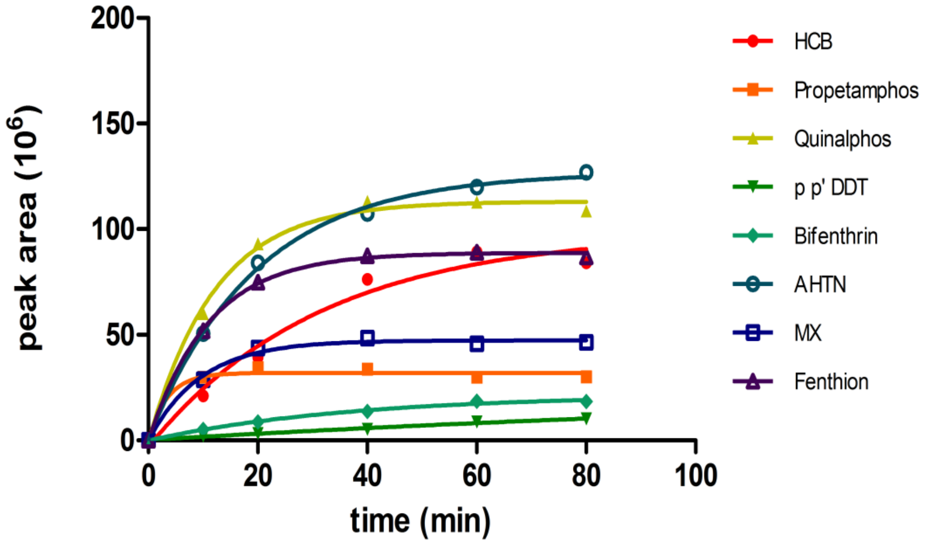

Supplementary Fig. 11. Extraction kinetic curves of contaminants by SPME. It could be observed that the extraction amounts of the whole contaminants reached a platuea after 80min. Thus, the 80 min extraction time was selected for the equibirium SPME studies.

Supplementary Table

Supplementary Table 1. The contaminants used in current study

Compound	Abbreviation	Category
Propetamphos	/	Organophosphorus pesticide
Quinalphos	/	Organophosphorus pesticide
Fenthion	/	Organophosphorus pesticide
Bifenthrin	/	Pyrethroid
Hexachlorobezene	HCB	Organochlorine pesticide
p-p' Dichlorodiphenyl Trichloroethane	p-p' DDT	Organochlorine pesticide
Tonalid	AHTN	PPCPs
Musk xylene	MX	PPCPs

Supplementary Table 2. Free concentrations (ng ·mL^{-1}) of the spiked irrigated water

	C_{free}
HCB	4.3 ±0.2
Propetamphos	26.0 ±5. 5
Quinalphos	27. 6 ±4. 4
p-p' DDT	3.2 ±0.9
Bifenthrin	22.9 ±5.1
AHTN	26.8 ±0. 9
MX	31.2 ±4.7
Fenthion	18.2 ±3.6

Supplementary Table 3. *In vivo* sampling rates (R_s, mg min^{-1}) and LOD (ng g^{-1})

	R_s	LOD
HCB	0.05±0.01	31.2
Propetamphos	1.03±0.37	0.3
Quinalphos	0.09±0.03	0.8
p-p' DDT	0.05±0.01	21. 6
Bifenthrin	0.01±0.002	34.8
AHTN	2.1±0. 50	0.3
MX	0.18±0.05	0.4
Fenthion	0.42±0.16	0.1

Supplementary Table 4. The physicochemical properties of soil used for plant growth

	Cultivated soil
Organic matter %	5.2
Total nitrogen content %	0.5
Total phosphorus anhydride content%	0.1
Water content %	<40
PH	5-6
EC (ds/m)	2.0
